# Introns as Gene Regulators: A Brick on the Accelerator

**DOI:** 10.3389/fgene.2018.00672

**Published:** 2019-02-07

**Authors:** Alan B. Rose

**Affiliations:** Department of Molecular and Cellular Biology, University of California, Davis, Davis, CA, United States

**Keywords:** intron, gene expression, transcription, gene regulation, promoter, intron-mediated enhancement

## Abstract

A picture is beginning to emerge from a variety of organisms that for a subset of genes, the most important sequences that regulate expression are situated not in the promoter but rather are located within introns in the first kilobase of transcribed sequences. The actual sequences involved are difficult to identify either by sequence comparisons or by deletion analysis because they are dispersed, additive, and poorly conserved. However, expression-controlling introns can be identified computationally in species with relatively small introns, based on genome-wide differences in oligomer composition between promoter-proximal and distal introns. The genes regulated by introns are often expressed in most tissues and are among the most highly expressed in the genome. The ability of some introns to strongly stimulate mRNA accumulation from several hundred nucleotides downstream of the transcription start site, even when the promoter has been deleted, reveals that our understanding of gene expression remains incomplete. It is unlikely that any diseases are caused by point mutations or small deletions that reduce the expression of an intron-regulated gene unless splicing is also affected. However, introns may be particularly useful in practical applications such as gene therapy because they strongly activate expression but only affect the transcription unit in which they are located.

## Introduction

It is crucial for the proper development and function of an organism that only some of the genes in its genome are expressed at a certain time, under particular conditions, or in each specific cell type. It is therefore fitting that a vast amount of research has been devoted to understanding how genes are regulated. A key control point in regulating expression is the initiation of transcription, and much is known about how the transcription machinery assembles near the site of initiation and starts transcribing (Luse, 2014; Robinson et al., 2016). Even though the textbook description of gene regulation by general and regulatory transcription factors binding to conserved DNA sequences in promoters^1^ and enhancers nicely explains the behavior of most genes, some observations are difficult to reconcile with the standard model. One example is the surprisingly large effect that some introns have on gene expression. In fact, certain introns may be the primary element directing the expression of some of the most highly expressed genes in the genome, causing the gene to be constitutively activated like a car with a heavy brick on its accelerator. These introns reveal a broad gap in our understanding of gene expression, and could be powerful tools for maximizing protein production in biotechnological and therapeutic applications. This article focuses on the specific kind of intron that increases mRNA accumulation because these introns seem to play a major role in regulating the gene in which they are located and because their effects are difficult to fit into our current understanding of gene expression.

There are numerous other important ways in which introns increase gene expression through general effects of splicing or specific features of individual introns acting by known mechanisms, as detailed in other reviews (Le Hir et al., 2003; Laxa, 2016; Shaul, 2017). Multiple interconnections between the various machineries that carry out splicing, transcription, polyadenylation, mRNA export, and translation provide opportunities for synergistic interactions through which introns can help generate more gene product (Maniatis and Reed, 2002; Dahan et al., 2011). These effects should apply to all efficiently spliced introns more or less equally if they are processed by the same splicing machinery. In addition to these general effects, specific introns may contain one or more various features that boost expression, such as an enhancer element (Kim et al., 2006) or sequences that increase translation (Akua and Shaul, 2013). Other introns are known to have direct or indirect negative effects on gene expression (Gromak, 2012; Jin et al., 2017). Because most eukaryotic genomes contain thousands of introns, there are multiple opportunities for introns to affect expression in a host of different ways. An enormous amount of work will be required to sort out all the mechanisms through which introns affect expression and the evolutionary relationships between them. Much of the existing research on introns has been performed in plants, which are the focus of this article. One possible reason is the relatively large number and small size of plant introns, which facilitates gene construction as well as computational analyses of intron sequences. Another is the ease of generating transgenic plants containing single-copy integrated genes, which matters because the observed effect of introns on gene expression is roughly an order of magnitude larger in stable transformants than it is when the same genes are used in transient expression assays (Rollfinke et al., 1998; Plesse et al., 2001). The phenomenon clearly is not limited to plants and the diverse range of organisms in which introns have been shown to elevate expression (Okkema et al., 1993; Duncker et al., 1997; Nott et al., 2003; Juneau et al., 2006; Goebels et al., 2013) suggests that the ability of introns to influence gene expression is either very ancient or arose multiple different times.

## mRNA-Increasing Introns

Certain introns located in transcribed sequences near the 5′ end of a gene have a large effect on mRNA accumulation (Callis et al., 1987). This has been described as “intron-mediated enhancement” or IME (Gallegos and Rose, 2015), although the need for a more specific name is becoming increasingly apparent as new information emerges, and to avoid confusion with the general use of the same phrase to describe any boost in expression caused by an intron regardless of mechanism (Mascarenhas et al., 1990; Laxa, 2016). Efficiently spliced introns vary widely in their effect on mRNA levels (Rose, 2002), indicating that the mechanism through which introns influence mRNA accumulation is not simply a function of splicing.

The main evidence that mRNA-increasing introns represent a new kind of regulatory element is that their properties are different from the characteristics of enhancers and promoters (Zabidi and Stark, 2016; Medina-Rivera et al., 2018). Experiments in which the location of an expression-stimulating intron was varied in a gene revealed that the intron must be within transcribed sequences and less than 1 kb from the start of transcription to increase mRNA levels (Rose, 2004; Gallegos and Rose, 2017). These introns are therefore unlike enhancer elements, which operate over long distances in both directions to activate transcription from a promoter (Zabidi and Stark, 2016). They are also unlike promoters in that the introns must be downstream of the transcription start site to affect expression. Deletion analysis, which has been used to locate important promoter and enhancer sequences, has proven largely ineffective in identifying the intron sequences responsible for increasing mRNA accumulation. In at least the case of the Arabidopsis *UBQ10* intron, this is because the active sequences are distributed throughout the stimulating intron rather than forming a single discrete element such as the binding site for a transcription factor (Rose et al., 2008).

## The Imeter Algorithm

**FIGURE 1:**
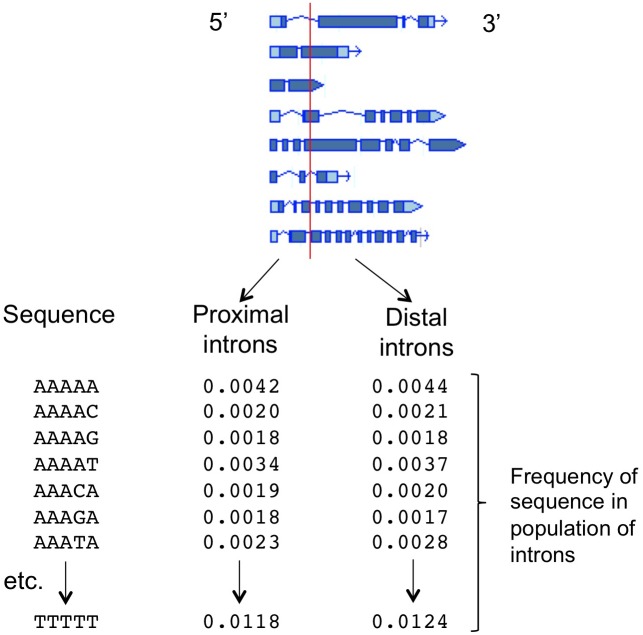
The function of the IMEter algorithm. The sequences of the introns in a genome are computationally separated into two groups based on whether the start of the intron is less than or greater than a threshold distance from the start of transcription for that gene. For each population of intron sequences, the frequency of occurrence of all possible nucleotide words of a given length (such as the pentamers shown) is calculated. A test sequence is compared to those two profiles, generating a numerical score that reflects the degree to which that sequence more strongly resembles the profile of promoter-proximal introns. Detailed descriptions of the underlying calculations, and the refinements added in different versions of the IMEter, can be found in Rose et al. (2008) and Parra et al. (2011).

The ability to predict which introns will increase mRNA accumulation, and to identify the intron sequences responsible for affecting expression, was greatly improved by the development of a computational tool known as the IMEter (Rose et al., 2008; Parra et al., 2011). This algorithm is based on the hypothesis that many introns throughout the genome might boost mRNA accumulation only when near the start of transcription, and as a result there may be detectable differences between promoter-proximal and promoter-distal introns caused by an increased abundance of IME-related sequences in promoter-proximal introns. The IMEter computationally separates all the introns in a genome into those that are near to, and those that are far from, the start of the gene in which they are found, with adjustable thresholds for “near” and “far” (Figure 1). The composition of all the introns in both groups is determined by calculating the frequency of occurrence of all possible nucleotide words of a given length, such as pentamers. A test sequence is then compared to these two k-mer profiles and a numerical score is generated, with a higher score reflecting a greater degree of similarity of that sequence to promoter-proximal introns. The algorithm works best for organisms with relatively small introns, and online versions are available for nearly three-dozen species of plants^2^. Computational difficulties prevent the development of IMEters for organisms such as mammals with very large introns. However, other approaches have begun to yield information about expression-stimulating sequences in human introns (Cenik et al., 2010).

## Genes Regulated by Introns Tend to Be Highly and Broadly Expressed

The strong correlation between the IMEter score of an intron and its ability to increase mRNA accumulation (Rose et al., 2008) supports the idea that the IMEter is detecting sequences in promoter-proximal introns that boost expression. It also allows the effect on expression of an intron to be predicted from its sequence alone. This in turn permits a broader analysis of the types of genes that contain introns likely to affect their expression, as well as the nature of the gene regulation that introns exert. A rough estimate based on the number of introns with high IMEter scores suggests that the expression of perhaps 10–15% of genes is influenced by an intron in plants, where the most computational work has been done (Gallegos and Rose, 2015).

Several lines of evidence indicate that introns drive a constitutive high level of expression in most or all tissues. Genome-wide, genes containing introns with high IMEter scores are generally expressed in a greater number of plant organs than genes without a high-scoring intron (Parra et al., 2011). This is in agreement with the kinds of genes in which stimulating introns historically have been discovered by comparing the expression of cDNA and genomic versions of the same gene. While this represents a small sample rather than an exhaustive survey, and there are multiple exceptions, many of the genes that contain a stimulating intron encode proteins that are needed in large amounts in most cell types, such as ubiquitin (Norris et al., 1993; Plesse et al., 2001), actin (Weise et al., 2006; Jeong et al., 2009), tubulin (Jeon et al., 2000; Fiume et al., 2004), ribosomal proteins (Chung and Perry, 1989; Ares et al., 1999), or elongation factors (Curie et al., 1991; Zhang et al., 2016). Additional evidence that introns generally produce strong constitutive gene expression is that inserting an expression-stimulating intron into a gene that is normally active only in certain cell types can override the regulation provided by the promoter and result in widespread expression (Jeong et al., 2006; Emami et al., 2013). The presence of a stimulating intron need not necessarily always result in ubiquitous expression because additional kinds of regulation could be combined with intron-driven expression. For example, a gene that contains a stimulating intron might be highly transcribed in all tissues, but the presence of a miRNA could eliminate the mRNA in certain cells, resulting in differential accumulation of mRNA in various locations.

**FIGURE 2:**
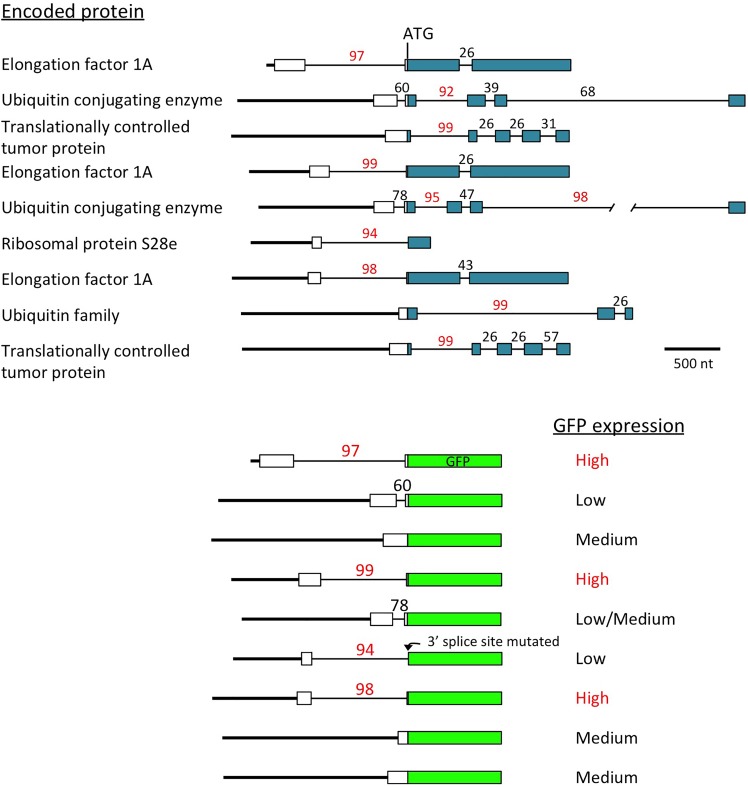
Introns may regulate the most highly expressed genes. Top: scale diagrams of the genes identified by Zhang et al. (2015) as producing the most mRNA throughout soybeans, aligned on their translational start codons. White rectangles indicate 5′-UTRs, colored boxes represent coding sequences, thick lines are promoters and thin lines are introns. IMEter scores (http://korflab.ucdavis.edu/cgi-bin/IMEter\_2014/web-imeter2.1.pl) are shown above each intron as a percentile. Bottom: the promoter of each gene was fused to GFP at the start codon, and the GFP activity produced by each construct is described (Zhang et al., 2015). One intron very close to the start codon had its 3′ splice site mutated by the restriction site created to make the fusion, probably preventing GFP expression if the intron is not spliced or uses an alternative 3′ splice site in GFP.

Another indication of the powerful effect introns can have on expression came unexpectedly from a study of the promoters of the most active genes in soybeans (Zhang et al., 2015). All of the genes identified as producing the highest amount of mRNA throughout the plant contain an intron with a high IMEter score (92nd percentile or higher) near the start of the gene (Figure 2). When the activity of the promoter fragments extending to the translational start codon of these genes were tested using GFP fusions, only those in which the intron was located in the 5′-UTR, and thus was included in the construct, gave strong GFP expression (Zhang et al., 2015). The introns in the other highly expressed genes were downstream of the ATG, so were not in the promoter fragments used, and these constructs gave moderate or low GFP expression. None of the genes identified in the same study that are highly expressed only in certain tissues contained an intron with a high IMEter score. While the role of introns in the expression of these genes has been only partially investigated (Zhang et al., 2016), these results suggest not only that introns are involved in activating the most strongly and widely expressed genes in the genome, but also that the intron may have a larger role than the promoter in driving this high level of expression.

## Introns Can Increase Expression in the Absence of a Promoter

The revolutionary idea that introns several hundred nucleotides downstream of the start of transcription can be more important than the promoter for generating abundant expression is supported by promoter deletion experiments in *Caenorhabditis elegans* and Arabidopsis. The ability of various versions of the *C. elegans unc-54* gene (encoding a myosin) to complement an *unc-54* mutant revealed that removing the introns from a genomic construct causes a much more severe reduction in gene function than does deleting all of the promoter except 8 nt of 5′-UTR (Okkema et al., 1993). Transcription in the promoterless version initiates in the plasmid sequences brought near to *unc-54* coding sequences by the deletion, suggesting either that the bacterial sequences fortuitously have promoter activity in nematodes or that intron sequences within the body of the gene cause transcription to initiate upstream of themselves.

Support for the idea that introns can regulate transcript initiation came from similar experiments in Arabidopsis. Deleting a 303 nt region that encompasses every known transcription start site and all but 18 nt of 5′-UTR of the Arabidopsis *TRP1* promoter does not appreciably diminish the expression of *TRP1:GUS* reporter gene fusions that contain a stimulating intron within coding sequences (Gallegos and Rose, 2017). Transcription in these constructs initiates in normally untranscribed intergenic sequences that without the deletion would be roughly 300 nt upstream of the normal transcription start sites, but due to the deletion are the same distance upstream of the intron as when the promoter is intact. Furthermore, when the intron in *TRP1:GUS* constructs with an intact promoter is moved from coding sequences into the 5′-UTR, transcription initiates further upstream than normal. These results reveal a greater flexibility in the sequences that act as transcription start sites than would be expected if the site of initiation is determined solely by transcription factors binding to conserved motifs in the promoter. There is evidence that some transcripts initiate a few hundred base pairs upstream of first introns in addition to the mapped transcription start sites of human genes as well (Bieberstein et al., 2012). A greater role for introns than promoters in controlling transcription would explain why the expression of some genes in mammals absolutely depends on introns, rather than simply being boosted by them (Buchman and Berg, 1988).

## Future Directions

The most pressing need is to begin to gain some understanding of the mechanism through which introns increase mRNA accumulation. The intron sequences in the DNA apparently play a larger role than do the RNA transcripts produced from them (Rose et al., 2011), and high IMEter scores are found not just in introns but also 5′-UTRs and to a lesser degree coding sequences near the start of a gene (Parra et al., 2011). Introns are a particularly suitable location for any such signals because the RNA they encode is removed from the transcript and therefore cannot interfere with the stability, translation efficiency, or protein encoded by the mature mRNA. One particularly promising areas of research is to determine the identity of factors, if any, that interact with introns that stimulate expression but not with non-stimulating introns. These could include transcription factors that are unusual in that they activate transcription several hundred nucleotides upstream of themselves. It is also possible that IME signals located anywhere in the first kilobase of transcribed DNA are capable of establishing a chromatin state in all cells that encourages a high level of unregulated transcription, and that the sequences in which transcription can initiate are surprisingly variable. This hypothesis could be tested by investigating the structure of the chromatin surrounding stimulating introns. The main challenge here will be to differentiate between differences in histone modification or occupancy that are directly caused by the intron, rather than those that may be a consequence of higher expression brought about by the intron through mechanisms unrelated to chromatin structure. Any direct biochemical analysis of the effect of introns on transcription probably will need to be done in a non-plant organism because *in vitro* systems that perform splicing or transcription of individual nuclear genes have not been developed for plants.

## Introns and Human Health

It is unlikely that any specific disease is caused by a mutation that disrupts the ability of a stimulating intron to maintain a high level of expression of a gene. The intron sequences that affect mRNA accumulation are redundant and dispersed, so a point mutation or even a large deletion in the intron would probably not significantly reduce the expression of the gene unless splicing was disrupted. Indeed, if the gene depended on the intron for expression, a mutation that removed the intron or eliminated its effect would likely cause lethality if the gene product is required in large amounts in most cells as seems typical for intron-regulated genes. However, expression-stimulating introns could prove to be valuable tools in several health-related applications. Their ability to massively increase expression could be used to maximize the production of pharmaceutical proteins such as monoclonal antibodies or therapeutic enzymes. Furthermore, because introns must be in transcribed sequences to affect expression and their influence only extends a few hundred base pairs, they may have a much lower risk than enhancer elements of inadvertently activating genes near the site of integration in gene therapy applications (Liu et al., 2015).

## Summary

Conventional promoters, with transcription factor binding sites and enhancers, are well suited to limit the expression of most genes to particular times and certain cell types. For those relatively few genes whose product is usually needed in large amounts in all tissues, a stimulating intron might be the best tool to keep the gene always “on” at top speed.

## Author Contributions

The author performed all aspects of preparing the manuscript.

## Conflict of Interest Statement

The author declares that the research was conducted in the absence of any commercial or financial relationships that could be construed as a potential conflict of interest.

## Footnotes

1 The word “promoter” in its broadest sense includes all the sequences involved in controlling the expression of a gene. However, it is more commonly used to mean the sequences (up to a few kilobases) around and upstream of the transcription initiation site but no further downstream than the start of translation, and this is how it will be applied here.

2 http://korflab.ucdavis.edu/cgi-bin/IMEter_2014/web-imeter2.1.pl
